# Stromal structure remodeling by B lymphocytes limits T cell activation in lymph nodes of *Mycobacterium tuberculosis*–infected mice

**DOI:** 10.1172/JCI157873

**Published:** 2022-11-01

**Authors:** Lina Daniel, Nayan D. Bhattacharyya, Claudio Counoupas, Yi Cai, Xinchun Chen, James A. Triccas, Warwick J. Britton, Carl G. Feng

**Affiliations:** 1 Immunology and Host Defence Group, School of Medical Sciences, Faculty of Medicine and Health,; 2 Centenary Institute,; 3 Charles Perkins Centre, and; 4 Microbial Pathogenesis and Immunity Group, School of Medical Sciences, Faculty of Medicine and Health, The University of Sydney, Sydney, New South Wales, Australia.; 5 Guangdong Provincial Key Laboratory of Regional Immunity and Diseases, Department of Pathogen Biology, Shenzhen University School of Medicine, Shenzhen, China.; 6 The University of Sydney Institute for Infectious Diseases, The University of Sydney, Sydney, New South Wales, Australia.; 7 Department of Clinical Immunology, Royal Prince Alfred Hospital, Camperdown, New South Wales, Australia.

**Keywords:** Immunology, Infectious disease, Adaptive immunity, Mouse models, Tuberculosis

## Abstract

An effective adaptive immune response depends on the organized architecture of secondary lymphoid organs, including the lymph nodes (LNs). While the cellular composition and microanatomy of LNs under steady state are well defined, the impact of chronic tissue inflammation on the structure and function of draining LNs is incompletely understood. Here we showed that *Mycobacterium tuberculosis* infection remodeled LN architecture by increasing the number and paracortical translocation of B cells. The formation of paracortical B lymphocyte and CD35^+^ follicular dendritic cell clusters dispersed CCL21-producing fibroblastic reticular cells and segregated pathogen-containing myeloid cells from antigen-specific CD4^+^ T cells. Depletion of B cells restored the chemokine and lymphoid structure and reduced bacterial burdens in LNs of the chronically infected mice. Importantly, this remodeling process impaired activation of naive CD4^+^ T cells in response to mycobacterial and unrelated antigens during chronic tuberculosis infection. Our studies reveal a mechanism in the regulation of LN microanatomy during inflammation and identify B cells as a critical element limiting the T cell response to persistent intracellular infection in LNs.

## Graphical abstract

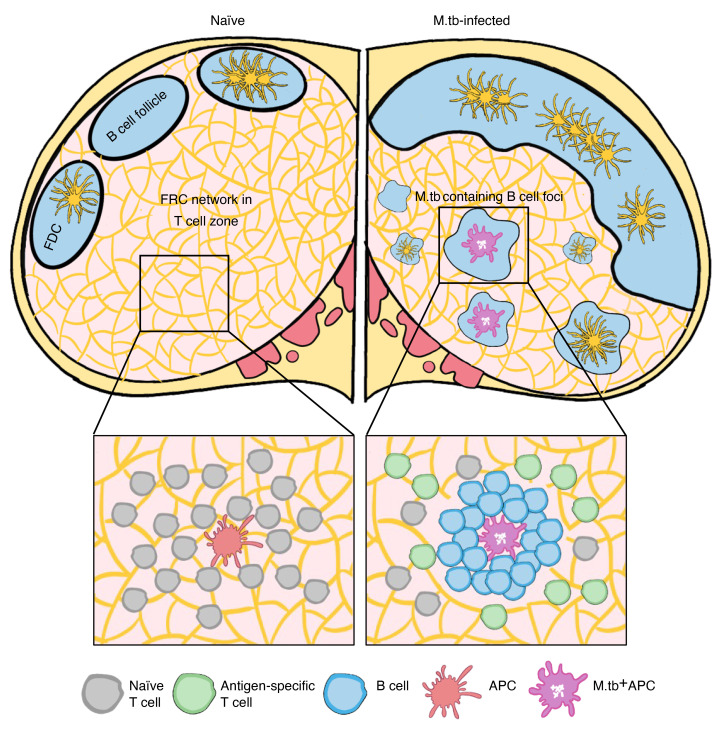

## Introduction

Lymph nodes (LNs) are highly compartmentalized secondary lymphoid organs in which adaptive immune responses are initiated. In the steady state, LNs are composed of 3 main compartments: B cell follicles, paracortex (also known as the T cell zone), and the medulla (1). The initiation of an efficient adaptive immune response relies on the strategic positioning of rare naive antigen-specific lymphocytes and their cognate antigen-presenting cells (APCs) within the LNs (2–4). This interaction is guided by a highly organized network of stromal cells that provide directional cues to mount an appropriate immune response (5–7).

The B cell area is maintained by follicular dendritic cells (FDCs) defined by their expression of complement receptors 1 (CR1 or CD35) and 2 (CR2 or CD21) (8). FDCs form stromal networks that maintain the survival, homing, and territoriality of B cells within the follicles (5). The paracortex is organized by ER-TR7–expressing fibroblastic reticular cells (FRCs) that secrete the chemokine ligands CCL19 and CCL21 (9–11). FRCs are not only critical in the maintenance of T cell territoriality but also guide interactions between APCs and their cognate T lymphocytes within the paracortex (2, 5, 12). The medulla is located at the basal area of the LNs and is rich in lymphatic vessels that allow lymphocytes to exit the LNs (1). Following initial CCL21-guided encounter of T cells and antigen-expressing APCs, activated T cells redistribute to peripheral regions of the LNs to complete the differentiation processes (2, 13, 14). Interfollicular regions located between B cell follicles, and the medullary ridge (also known as the deep paracortex), situated at the paracortical-medullary border, are 2 specialized areas involved in optimizing Th1 differentiation (15–18).

Transient downregulation of key homing chemokines and subsequent reorganization of secondary lymphoid structures are seen following acute bacterial infections, such as with *Listeria monocytogenes* (19), and viral infections, such as lymphocytic choriomeningitis (20) and cytomegalovirus (21), vaccinia virus, and influenza virus infection (19). Moreover, perturbation of LN structure and/or chemotactic cues has been shown to compromise CD8^+^ T cell priming (19) and B cell response against a secondary infection (22), as well as enhancing virulence of pathogens such as *Salmonella* (23). However, the impact of chronic infection on LN structure and function, including the activation of naive CD4^+^ T cells, remains unclear.

*Mycobacterium tuberculosis*, the intracellular bacterium that causes tuberculosis, is known to establish persistent infection in both humans and mice. Following aerosol infection, *M. tuberculosis* replicates logarithmically in the lungs until the onset of adaptive immunity, initiated in the lung-draining mediastinal lymph node (mLN) (24–26). Following the activation and differentiation of *M. tuberculosis*–specific CD4^+^ T cells in the mLN, effector Th1 cells migrate to the lungs to exert their effector functions for bacterial control (27–29). While a recent study has suggested that a significant proportion of *M. tuberculosis*–infected humans can eliminate the pathogen (30), *M. tuberculosis* is known to persist in infected mice (31, 32), indicating that, while necessary, the Th1 response may be suboptimal in this model. Extensive studies dissecting mechanisms of suboptimal CD4^+^ T cell immunity in mice have spawned multiple hypotheses. These include decreased *M. tuberculosis*–specific T cell numbers during chronic infection (33, 34), trapping of effector T cells in the lung vasculature (35), and suboptimal activation of effector CD4^+^ T cells in *M. tuberculosis* granulomas (36–38).

In contrast, few investigations have analyzed the lung-draining mLN in *M. tuberculosis* infection. These have mainly investigated bacterial numbers (39) and initial T cell activation in early stages following infection (24, 26). The impact of persistent *M. tuberculosis* infection on the lymphoid microenvironment and the CD4^+^ T cell response in the mLN is unknown. Using tissue imaging, cell depletion, and T cell adoptive transfer approaches, we investigated the spatial organization and T cell–activating function of the mLN during the early and later stages of *M. tuberculosis* infection in mice. Here we show that *M. tuberculosis*–infected mLN undergoes extensive structural changes driven by the disproportionate expansion of B lymphocytes. B cell invasion and development of ectopic CD35^+^ FDC clusters in the paracortex altered the position of CCL21-expressing FRCs and T cells. Importantly, depletion of B cells restored chemokine and lymphoid structure and reduced *M. tuberculosis* burdens in mLNs of the chronically infected mice. Moreover, this progressive remodeling led to significantly impaired CD4^+^ T cell responses to *M. tuberculosis* and an unrelated antigen. Together, these findings reveal previously unknown mechanisms regulating the LN microenvironment in inflammation and CD4^+^ T cell response to persistent infection in mice.

## Results

### Induction of regional immunity coincides with disproportionate B cell expansion in the lung-draining LNs of M. tuberculosis–infected mice.

**Figure 1:**
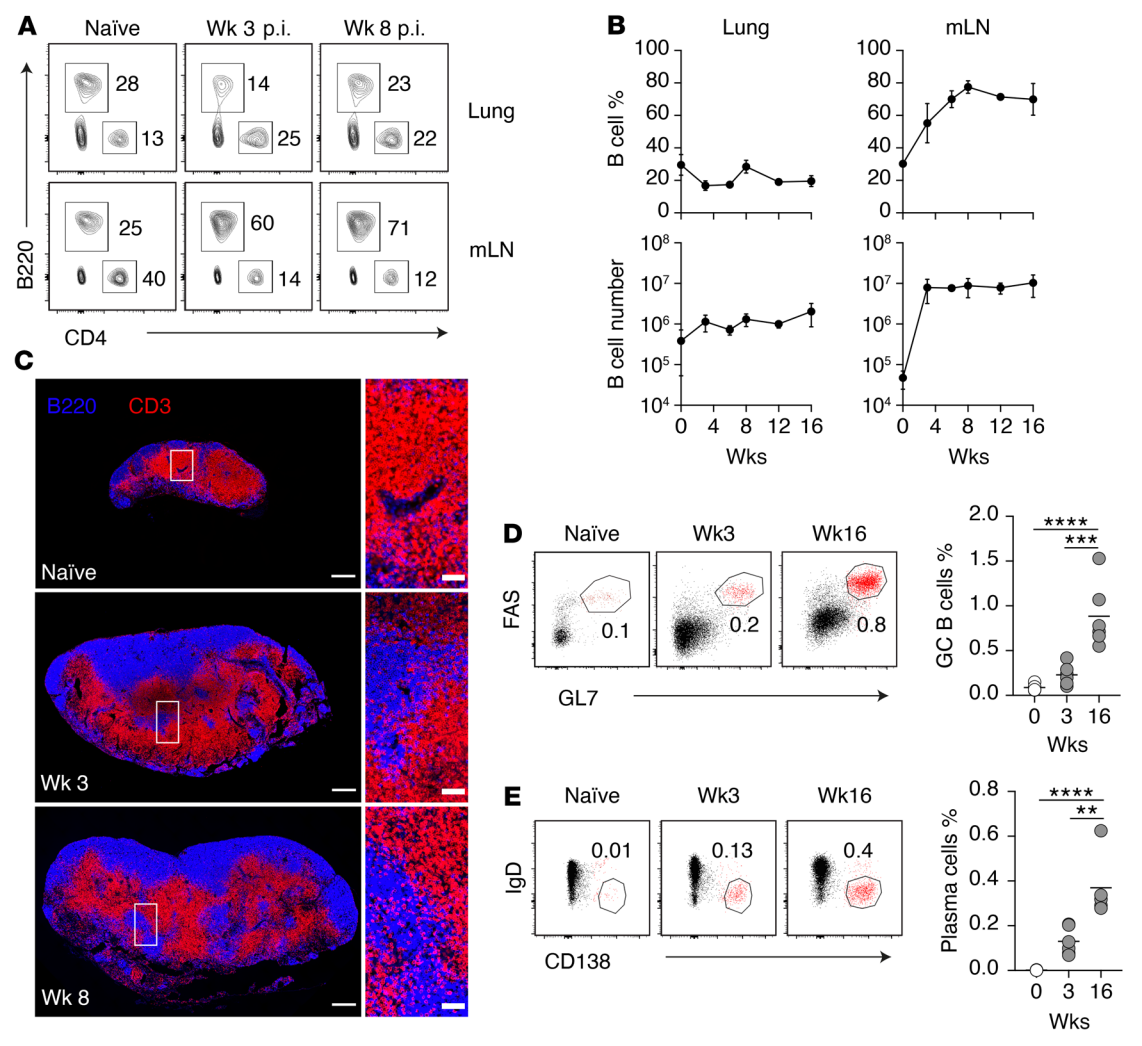
*M. tuberculosis* induces disproportional B cell expansion and altered lymphocyte localization in lung-draining LNs. (**A**) Representative FACS plots showing the percentage of B220^+^ and CD4^+^ cells in the lung and the mLN of naive and *M. tuberculosis*–infected C57BL6 WT mice. (**B**) Percentage and number of B220^+^ B cells in the lungs and mLN at the indicated time points. Data shown are the mean ± SD. (**C**) Representative images of mLNs from naive, week-3 infected, and week-8 infected WT mice. The tissues were stained for B220 to detect B cells and CD3 to detect T cells. Scale bars: 300 μm for whole LN images (left), 50 μm for enlarged regional images (right; boxed areas in the left images). (**D** and **E**) Representative flow cytometry plots and summary data of germinal center B cells (GL7^+^FAS^+^) gated on CD19^+^B220^+^CD138^–^IgD^lo^ cells (**D**) and plasmablast B cells (IgD^–^CD138^+^) gated on CD19^+^B220^lo^ cells (**E**) in the mLNs at the indicated time points. Data in **A**–**C** are representative of more than 5 independent experiments with similar results (*n* = 5 mice per group). Data in **D** and **E** are representative of 3 independent experiments with similar results (*n* = 5 mice per group). Statistical differences between groups were determined using 1-way ANOVA with Tukey’s multiple-comparison test. **P < 0.01, ***P < 0.001, ****P < 0.0001.

To understand how regional immunity is regulated in the mLN, we first analyzed the major lymphocyte populations in early (week 3 postinfection [p.i.]) and late (week 8 p.i.) infection stages. Flow cytometric analysis showed that *M. tuberculosis* infection significantly altered the T/B lymphocyte ratio in the mLN beginning from week 3 p.i. (Figure 1A), coinciding with the initiation of T cell response (24, 25, 28). While the proportion and number of B cells in the lungs did not change (Figure 1, A and B), *M. tuberculosis* induced a gradual increase in B cells in the infected LNs compared with their naive counterparts, which plateaued between weeks 4 and 8 p.i., representing a 1,000-fold increase in B lymphocyte numbers (Figure 1B). To determine whether *M. tuberculosis* infection and B cell expansion alter the specialized lymphoid organization in the mLN, we next compared the localization of T and B lymphocytes in the mLN of naive and infected mice. Immunohistologic analysis revealed that in naive mLN, CD3^+^ T cells were localized in the paracortical region whereas B220^+^ B cells were confined to the cortical regions and separated into distinct follicles (Figure 1C and Supplemental Figure 1A; supplemental material available online with this article; https://doi.org/10.1172/JCI157873DS1). Moreover, in addition to changing LN size, *M. tuberculosis* infection led to marked reorganization of the mLN. B cell follicles were enlarged and became fully or partially connected. B and T lymphocytes were no longer compartmentalized into distinct intranodal regions. Importantly, B cells invaded into the paracortex, traditionally occupied by T lymphocytes, and appeared to cause the displacement of T cell populations. These findings reveal a previously unrecognized B cell–driven tissue remodeling process occurring in the lung-draining mLN of pulmonary *M. tuberculosis*–infected mice.

To address the consequence of B cell expansion within the mLN, we assessed the humoral response to *M. tuberculosis* infection. While we observed that disproportionate B cell expansion began from 3 weeks and persisted until 16 weeks p.i., the percentage of germinal center B cells (GL7^+^FAS^+^) and plasmablasts (IgD^–^CD138^+^) (Figure 1, D and E) did not increase significantly until the chronic stage of the infection when compared with naive and week 3 p.i. A similar trend was also observed for B cell populations expressing the proliferation marker Ki-67 (Supplemental Figure 1B), suggesting that accumulation of B cells in the mLN cannot be explained fully by increased local proliferation. Immunohistologic analysis revealed that germinal centers, identified using GL7 and Ki-67, were formed in both naive and *M. tuberculosis*–infected mice (Supplemental Figure 1C). In addition, we analyzed serum IgG antibody responses to *M. tuberculosis* culture filtrate protein (CFP) and whole-cell lysate (WCL) before and kinetically after infection (weeks 3, 8, and 10) (Supplemental Figure 2, A and B). We found that infection-induced total IgG and IgG2c responses appeared to be transient and most apparent in week 3 infected mice, although total IgG response to WCL at week 3 was not statistically significant compared with preinfection levels (Supplemental Figure 2). IgG1 response to either bacterial preparation was undetected over the course of 10-week infection. Together, these findings suggest that the high level and sustained accumulation of B cells in mLN cannot be explained fully by increased local proliferation, and the function of B cell expansion at this site is likely independent of antibody production.

### B cell expansion alters the organization of CCL21-secreting FRC network.

**Figure 2:**
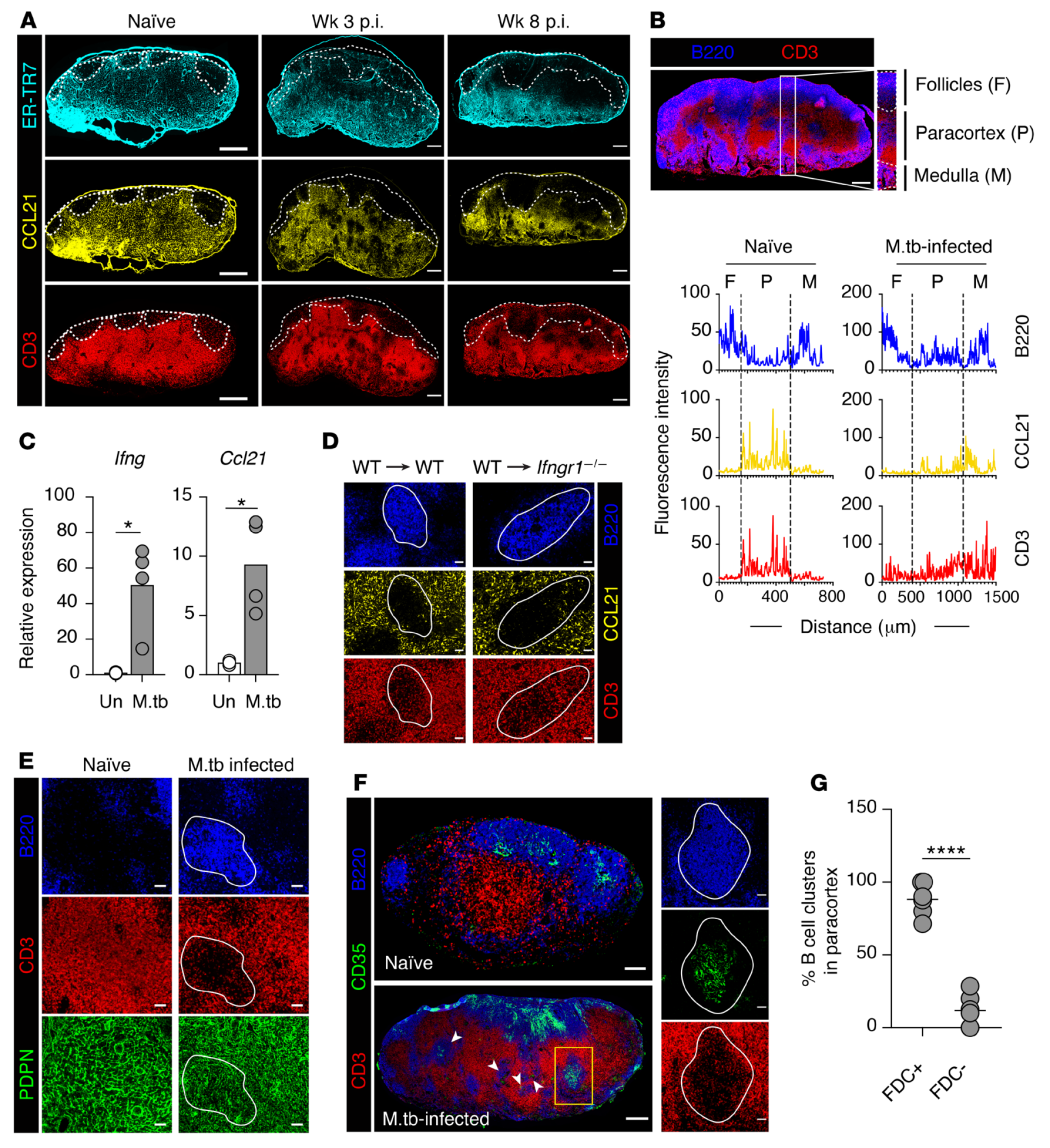
B cell expansion disturbs stromal cell organization in *M. tuberculosis*–infected mLN. (**A**) Images of mLN sections from naive and infected mice. Dashed line delineates the B cell follicles (staining not shown). Scale bars: 300 μm. (**B**) Representative image of B220- and CD3-stained week 8 *M. tuberculosis*–infected mLN. Scale bar: 300 μm. Boxed area represents the cortex-medullary axis. Scale bar: 50 μm. The histograms represent the average fluorescence intensity of each marker along the cortex/medullary axis of the mLN. Dashed line: boundary between the follicles, paracortex, and medulla. (**C**) *Ifng* and *Ccl21* expression in uninfected and 8-week-infected LNs measured by qRT-PCR. Data are the mean fold increase (*n* = 4) ± SD relative to nondraining inguinal LN. (**D**) mLN images of 8-week *M. tuberculosis*–infected chimeric mice (WT or *Ifngr^–/–^* recipients reconstituted with WT bone marrow cells). White line: paracortical B cell cluster boundary. Scale bars: 50 μm. (**E**) Images of B220-, CD3-, and PDPN-stained mLN from naive and 8-week *M. tuberculosis*–infected mice. White line: paracortical B cell cluster boundary. Scale bars: 50 μm. (**F**) mLN sections from naive and 8-week *M. tuberculosis*–infected mice. Arrowheads indicate CD35^+^ FDC-containing paracortical B cell clusters in infected mLN. Expression of B220, CD3, or CD35 in a paracortical B cell cluster (boxed region) in *M. tuberculosis*–infected mLN is shown at right. Scale bars: 100 μm (left) and 50 μm (right). (**G**) Quantification of paracortical B cell clusters with or without CD35^+^ cells in infected mLN. Data in **A**, **B**, and **D**–**F** are representative of 2 independent experiments with similar results (*n* = 4 mice per group), and data in **G** are from 2 experiments (*n* = 6 mice). Statistical differences between groups were determined using Student’s *t* test. *P < 0.05, ****P < 0.0001. M.tb, *M. tuberculosis*.

The initiation of an efficient immune response depends on the highly structured microanatomy of the LNs maintained by the stromal reticular network (7, 40, 41). The changes in lymphocyte localization suggested that the stromal cell architecture might be modified following *M. tuberculosis* infection. Given the critical importance of FRCs in secreting CCL19 and CCL21 and maintaining the territoriality of T cells (5, 10), we first examined the stromal network by staining for the FRC-specific marker ER-TR7, a secreted extracellular matrix component (42, 43), as well as for the paracortical T cell–homing chemokine CCL21. In naive mice, the ER-TR7^+^ FRC network appeared reticular and uniformly distributed throughout the paracortical region of the mLN (Figure 2A). A similar distribution pattern was also observed for the chemokine CCL21, with the position of CD3^+^ T lymphocytes precisely reflecting the distribution of the CCL21-expressing stromal network (Figure 2A). The distinct expression patterns of FRCs and CCL21 delineated the paracortex in the LNs of naive mice. In contrast, in *M. tuberculosis*–infected mice ER-TR7 and CCL21 expression became sporadic. This expression pattern was mirrored by the uneven distribution of CD3^+^ T lymphocytes in the paracortex (Figure 2A).

Given our observation of the invasion of B cells into the paracortex in *M. tuberculosis*–infected mLN, we next determined the intranodal distribution of CCL21-, CD3-, and B220-expressing regions by quantifying the fluorescence intensity of respective markers along the cortex and medulla (identified by the expression of the lymphatic vessel marker Lyve-1 [ref. 44]; data not shown) on consecutive sections (Figure 2B, top panel). As expected, in naive mLN, B220 signal was detected mainly within the follicles and medullary region of the LN, with minimal signal within the paracortex. In contrast, CCL21 and CD3 signals were detected primarily in the paracortex (Figure 2B, bottom panel), consistent with previous reports showing T cell localization in CCL21-rich areas in LNs draining other tissue sites (9, 10, 45). However, following *M. tuberculosis* infection, B220 signal was no longer exclusively localized within the cortical follicles, as substantial B220 fluorescence signals were detected within the paracortex. Importantly, the increased B220 signal was correlated negatively with CCL21 and CD3 expression, suggesting that T and B cells are positioned in a mutually exclusive manner in the *M. tuberculosis*–remodeled mLN.

Dysregulated CCL21 chemokine expression in secondary lymphoid tissues has been previously linked to several distinct mechanisms, including IFN-γ–mediated suppression and stromal cell destruction (19, 21, 23, 46). Since IFN-γ is strongly induced by *M. tuberculosis* (27), we examined whether it is responsible for the observed CCL21 downregulation. However, we found that mRNA expression of both *Ifng* and *Ccl21* in bulk tissue was upregulated relative to uninfected LNs (Figure 2C). Since CCL21 is mainly produced by non-hematopoietic cells, we generated chimeric mice using WT bone marrow cells with either irradiated *Ifngr^+/+^* or *Ifngr^–/–^* mice as recipients. By examining regions within the paracortex populated with B cells in the infected chimeric mice, we observed a similar increase in B cells and loss of CCL21 expression in both groups (Figure 2D). These results indicate that the intermittent expression of CCL21 by stromal cells within the mLN is independent of IFN-γ signaling.

Next, we investigated the effect of B cell expansion on stromal network integrity using a broad stromal cell marker, podoplanin (PDPN), which is expressed on the majority of stromal cell populations in LNs, including FRCs and FDCs (47). Following infection, the PDPN^+^ reticular network was found to remain intact, excluding the possibility of stromal cell structure damage. However, we observed that the stromal cells appeared to be denser compared with those in naive mLN (Figure 2E). This increased density likely accommodates the dramatic increase in LN size following infection (48). Interestingly, the reticular pattern of PDPN cells was preserved following infection in areas occupied by T cells but appeared sparse and filamentous within paracortical B cell–populated regions (Figure 2E). This raised the question of whether the paracortical stromal cells occupying B cell–rich areas are typical FRCs.

Given the expansion of B cells, we next examined B cell–specific stromal cells. Along with FDCs, 2 other stromal cell populations, termed versatile stromal cells (VSCs) and marginal reticular cells (MRCs), have been shown to support expanding B cell follicles following inflammation in mice (49–51). Upon inflammation, MRCs are triggered by expanding follicular B cells to differentiate into typical FDCs (50). In contrast, VSCs do not differentiate into typical FDCs, but do begin to exhibit FDC functional properties upon interaction with B cells (49). To examine the changes in the B cell–associated stromal network, naive and *M. tuberculosis*–infected mLNs were stained with anti-CD35. As anticipated, in naive mLN, CD35^+^ FDCs were confined to the cortical follicles. Following infection, we observed that along with B cells, FDCs extended and crossed into the T–B cell border (Figure 2F). Interestingly, most B cell clusters localized within the paracortex contained CD35^+^ cells (Figure 2, F and G). These CD35^+^ stromal cells are likely to have been MRCs that were triggered to exhibit FDC properties via interaction with expanding B cells. Together, these data suggest that the translocation of CD35^+^ FDC clusters and B cells into the paracortex following infection contributes to the displacement of CCL21-expressing FRCs and T cells in this region.

### B cell depletion restores FRC and T cell organization and reduces M. tuberculosis loads in mLN.

**Figure 3:**
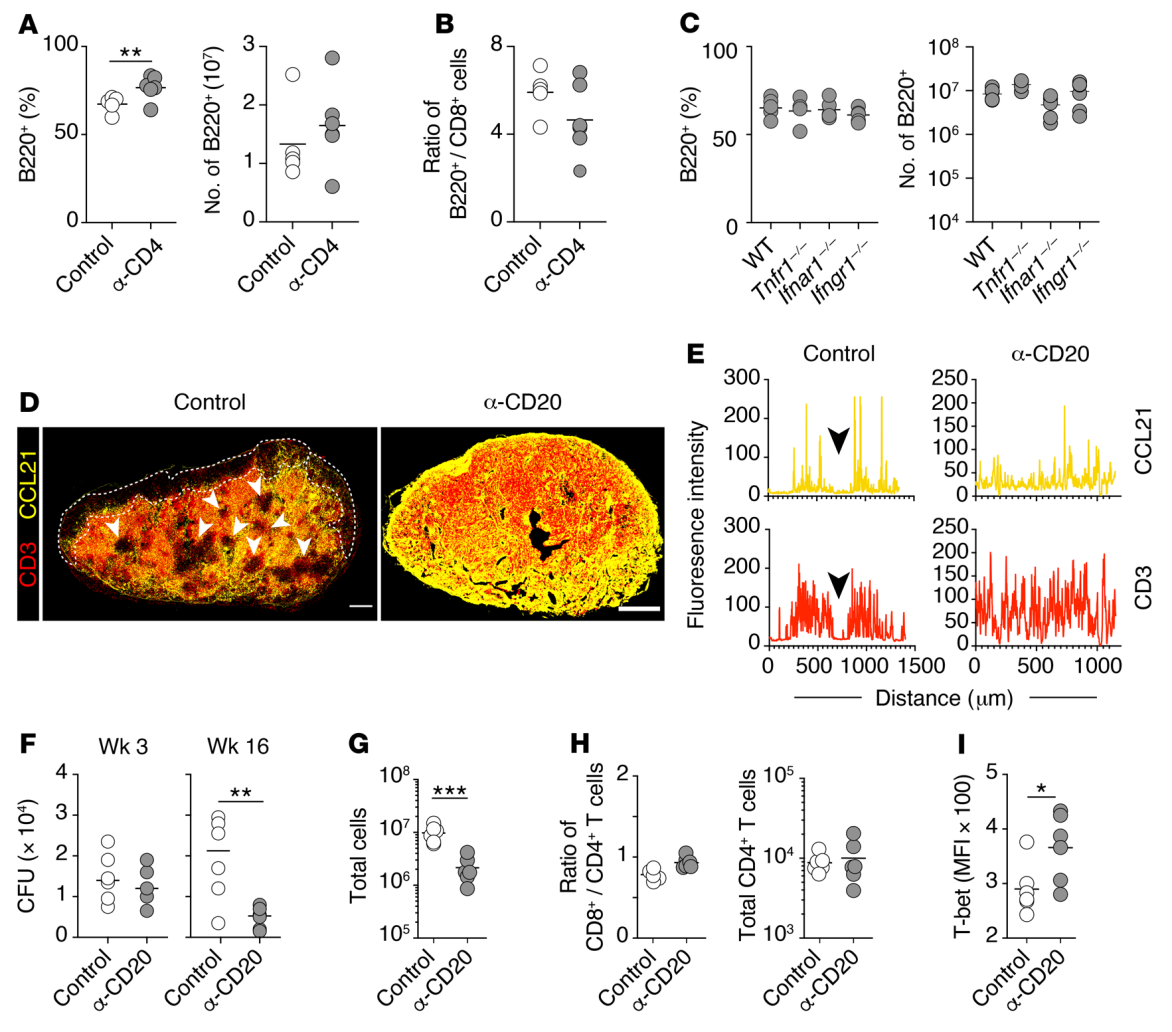
B cell depletion restores T cell organization and reduces *M. tuberculosis* loads in mLN. (**A** and **B**) Summary flow cytometry data of mLN from anti-CD4 mAb–treated B6 WT mice at week 8 p.i. (**A**) Percentages and numbers of B220^+^ cells. (**B**) Ratio of B220^+^ cells to CD8^+^ T cells. (**C**) Proportion and numbers of B cells (B220^+^) in the mLN of week-3 *M. tuberculosis*–infected WT, *Tnfr1^–/–^*, *Ifnar1^–/–^*, and *Ifngr1^–/–^* mice. (**D**) Representative images of CCL21- and CD3-costained mLN from isotype control or anti-CD20 mAb–treated *M. tuberculosis*–infected mice. Scale bars: 300 μm. (**E**) Histograms representing the average fluorescence intensity of CCL21 and CD3 along the cortex-medullary axis of isotype control and anti-CD20–treated mice. Arrowheads represent paracortical region occupied by B cell clusters in control mice. (**F**) Bacterial loads in the mLN of control and anti-CD20 mAb–treated mice measured at weeks 3 and 16 p.i. (**G**) The total number of cells in the mLN of isotype control and anti-CD20–treated mice at week 16 p.i. (**H**) Ratio of CD4^+^ to CD8^+^ T cells (left) and total number of CD4^+^ T cells (right) in the mLN of isotype control and anti-CD20–treated mice at week 16 p.i. (**I**) MFI of expression in ESAT-6_4–17_–specific CD4^+^ T cells in the mLN at week 16 p.i. Data in **A**–**E** are representative 2 independent experiments (*n* = 6–8 mice per group). Data in **F**–**I** are representative of 3 independent experiments with similar results (*n* = 6 mice per group). Statistical differences between groups were determined using Student’s *t* test (**A**, **B**, and **F**–**I**) or 1-way ANOVA with Tukey’s multiple-comparison test (**C**). *P < 0.05, **P < 0.01, ***P < 0.001.

To understand the mechanisms underlying the remodeling of mLNs associated with B cell expansion, we investigated the role of major immune cell populations and cytokines commonly associated with this infection (27, 52). To assess the contribution of CD4^+^ T cell help in B cell expansion (53), we depleted CD4^+^ T cells in *M. tuberculosis*–infected mice using anti-CD4 depleting antibodies. Mice depleted of CD4^+^ T cells showed a marginal increase in the percentage and absolute numbers of B cells in the mLN compared with their istotype control–treated counterparts (Figure 3A). To confirm that these minor changes were due to the absence of CD4^+^ T cells and not specific to B cell populations, we compared the ratio of B cells to CD8^+^ T cells and found that this ratio was similar in both treated and control LNs (Figure 3B), indicating that B cell expansion is independent of CD4^+^ T cells. Similarly, the percentage and numbers of B cells in the mLN of the infected *Tnfr1^–/–^*, *Ifnar1^–/–^*, and *Ifngr1^–/–^* mice were comparable to those in WT mice (Figure 3C). Together, these findings indicate that the disproportionate increase in B cells in the mLN is mediated by a mechanism independent of the key players in the host response to *M. tuberculosis* infection: CD4^+^ T cells, TNF-α, and IFNs.

We next investigated whether B cell depletion could prevent the *M. tuberculosis*–induced remodeling processes in the mLN. Anti-CD20 treatment restored the homogeneous distribution pattern of CD3^+^ T cells, which mirrored the uninterrupted CCL21 expression throughout the paracortex (Figure 3, D and E, and Supplemental Figure 3, A and B). This demonstrates definitively that the altered distribution of CCL21 and CD3 is dependent on B cell expansion. We also examined mLNs’ *M. tuberculosis* burdens in mice that had received the anti-CD20 antibody treatment at the initiation or chronic phase of the infection (antibody injection started from either 1 day or 12 weeks after aerosol *M. tuberculosis* infection). We found that while similar at week 3, CFUs in the mLN of B cell–depleted mice were reduced significantly in comparison with control *M. tuberculosis*–infected mice at week 16 p.i. (Figure 3F), revealing a detrimental role for B lymphocytes in bacterial control during chronic *M. tuberculosis* infection. Moreover, depletion of B cells resulted in approximately 90% reduction in the total number of cells recovered from the infected LNs (Figure 3G and Supplemental Figure 4, A and B), confirming that B cells are the main contributors to mLN enlargement following infection. Nonetheless, the ratio of CD8^+^ to CD4^+^ T cells and the total number of CD4^+^ T cells remained comparable between control and anti-CD20–treated mice (Figure 3H), indicating that B cells do not regulate global lymphocyte recruitment into the mLN and the improved bacterial control is not due to a change in total CD4^+^ T cell numbers. Interestingly, T-bet expression level in ESAT-6–specific CD4^+^ T cells was significantly higher in the mLN of anti-CD20–treated animals than in the controls (Figure 3I), suggesting enhanced Th1 differentiation in the absence of B cells. Together, these data indicate that B cells impair bacterial control in the mLN and are responsible for the remodeling of T cell localization and chemokine expression patterns in infected mLN.

### B cells entrap M. tuberculosis–containing CD11b^+^ cells in the mLN of chronically infected mice.

**Figure 4:**
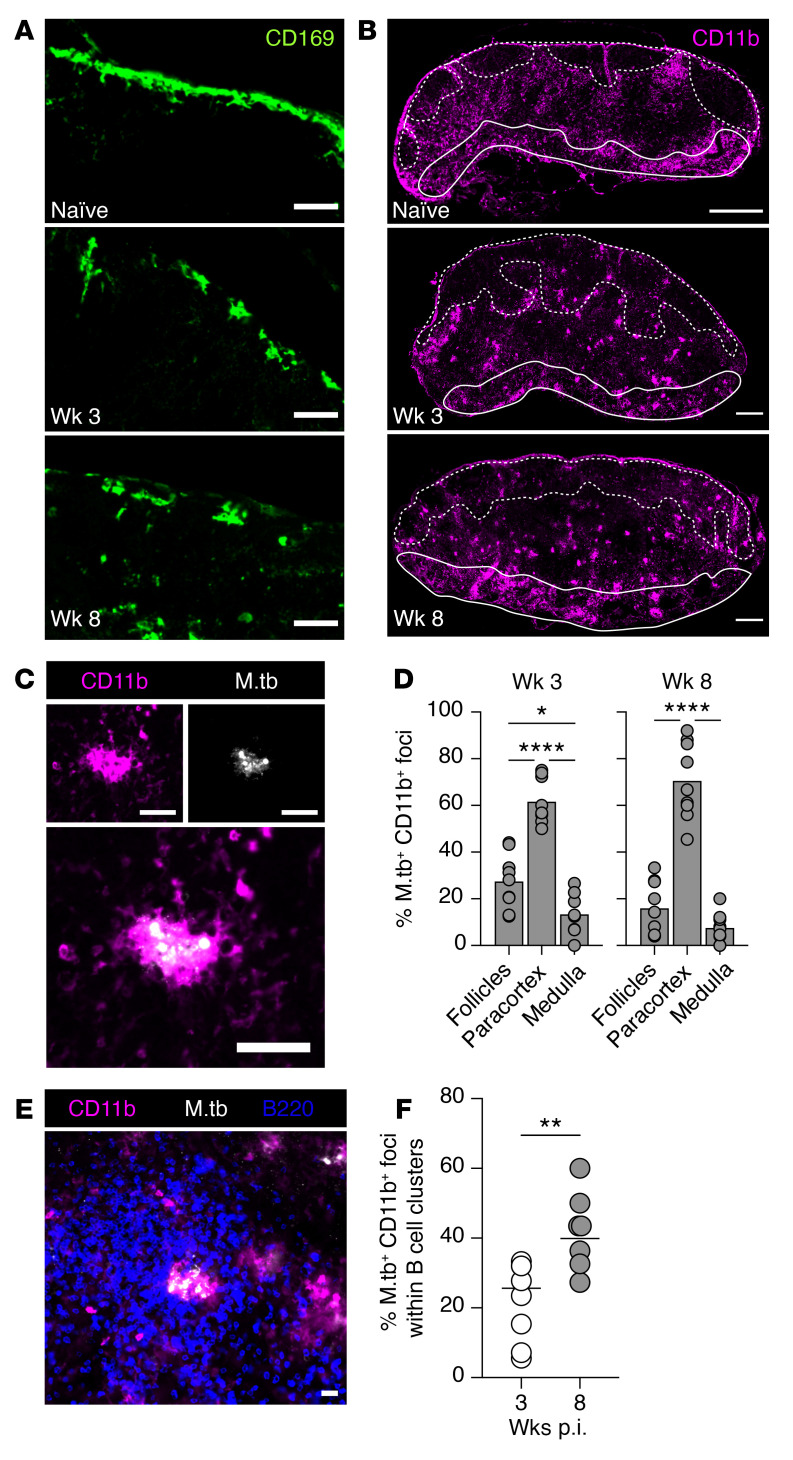
*M. tuberculosis* is mainly contained in CD11b^+^ myeloid cells within paracortical B cell clusters. (**A** and **B**) Immunofluorescence images of mLN from naive and week 3 and 8 *M. tuberculosis*–infected mice stained for CD169 (**A**) (scale bars: 50 μm) and CD11b (**B**) (scale bars: 300 μm). Dashed white line delineates B cell follicles, and solid white line defines the medulla. (**C**) Representative immunofluorescence staining of CD11b^+^ myeloid cells and *M. tuberculosis* bacteria. Scale bars: 50 μm. (**D**) Quantification of CD11b^+^*M. tuberculosis*^+^ foci in the subcompartments of the mLN at weeks 3 and 8 after *M. tuberculosis* infection. The analysis was performed on stitched images of entire mLN, and subcompartments of the mLN were defined as indicated in Figure 2B, top panel. (**E**) Representative image of CD11b^+^*M. tuberculosis*^+^ foci localized within B cell cluster in the paracortex. Scale bar: 50 μm. (**F**) Quantification of CD11b^+^*M. tuberculosis*^+^ foci within B cell clusters in the paracortex of whole mLN sections at weeks 3 and 8 after *M. tuberculosis* infection. Circles and bars denote the individual mLN sections and group means, respectively. Data shown in **D** and **F** are pooled from 3 independent experiments (*n* = 7–9 mice in total). Statistical differences between groups were determined using 1-way ANOVA with Tukey’s multiple-comparison test (**D**) or Student’s *t* test (**F**). *P < 0.05, **P < 0.01, ****P < 0.0001.

We next examined the tissue organization of major myeloid cell populations and their relationship with the expanding B cells in *M. tuberculosis*–infected mLN. Maintenance of the lymph-tissue interface by subcapsular sinus (SCS) CD169^+^ macrophages is critical to LN organization (22, 54, 55). Their strategic positioning allows them to screen and capture antigen draining from the lymphatics and prevent systemic dissemination (56). SCS macrophages are also involved in initiating humoral immune responses by presenting antigen to follicular B cells (54, 57). Following *M. tuberculosis* infection, we observed a marked disruption to the compact SCS layer seen in naive mLN (Figure 4A and Supplemental Figure 3C), which could be due to the increased influx of myeloid cells into draining LNs as suggested previously (22).

Since most *M. tuberculosis*–harboring myeloid cells in the mLN arrive from the lung through the afferent lymphatics (58), we investigated the distribution of CD11b-expressing myeloid cells. CD11b is expressed by migratory DCs and LN-resident DCs (cDC2, involved in CD4^+^ T cell priming) (59, 60). In naive mice, CD11b^+^ myeloid cells were dispersed throughout the paracortex and most noticeably in the interfollicular regions (IFRs; located between B cell follicles) and the medullary ridge (MR; located at the paracortical-medullary border) of the mLN (Figure 4B and Supplemental Figure 3D). However, at 3 weeks p.i., many CD11b^+^ myeloid cells clustered together and were distributed in all 3 regions of the mLN. This clustering organization and distribution persisted to at least week 8 p.i. Interestingly, some CD11b^+^ clusters were present within B cell follicles and near the SCS following infection, suggesting that they are likely migratory myeloid cells draining from the lung.

To identify the intranodal location of *M. tuberculosis*–infected cells, LN sections were stained with a polyclonal antibody against *M. tuberculosis* purified protein derivative (PPD). Consistent with previous studies (58), we found that *M. tuberculosis* was exclusively retained within CD11b^+^ myeloid cells (Figure 4C). At both week 3 and week 8 p.i., *M. tuberculosis*–containing myeloid cells were located mainly within the paracortex (Figure 4D). Since the paracortex becomes increasingly occupied by B cell aggregates throughout *M. tuberculosis* infection, we examined the localization of *M. tuberculosis*–containing CD11b^+^ foci in relation to B cells. By quantifying *M. tuberculosis*–containing myeloid foci confined within B cell clusters in the paracortex at the early and late stages of the infection, we observed that a substantial proportion of the infected foci were enclosed within B cell aggregates (Figure 4, E and F); by week 8 p.i., almost half of these foci were found to be enclosed within B cell clusters (Figure 4F), suggesting possible segregation of *M. tuberculosis*–containing infected myeloid cells by invading B cells.

### Reduced CD4^+^ T cell activation in the mLN during the later stage of M. tuberculosis infection.

**Figure 5:**
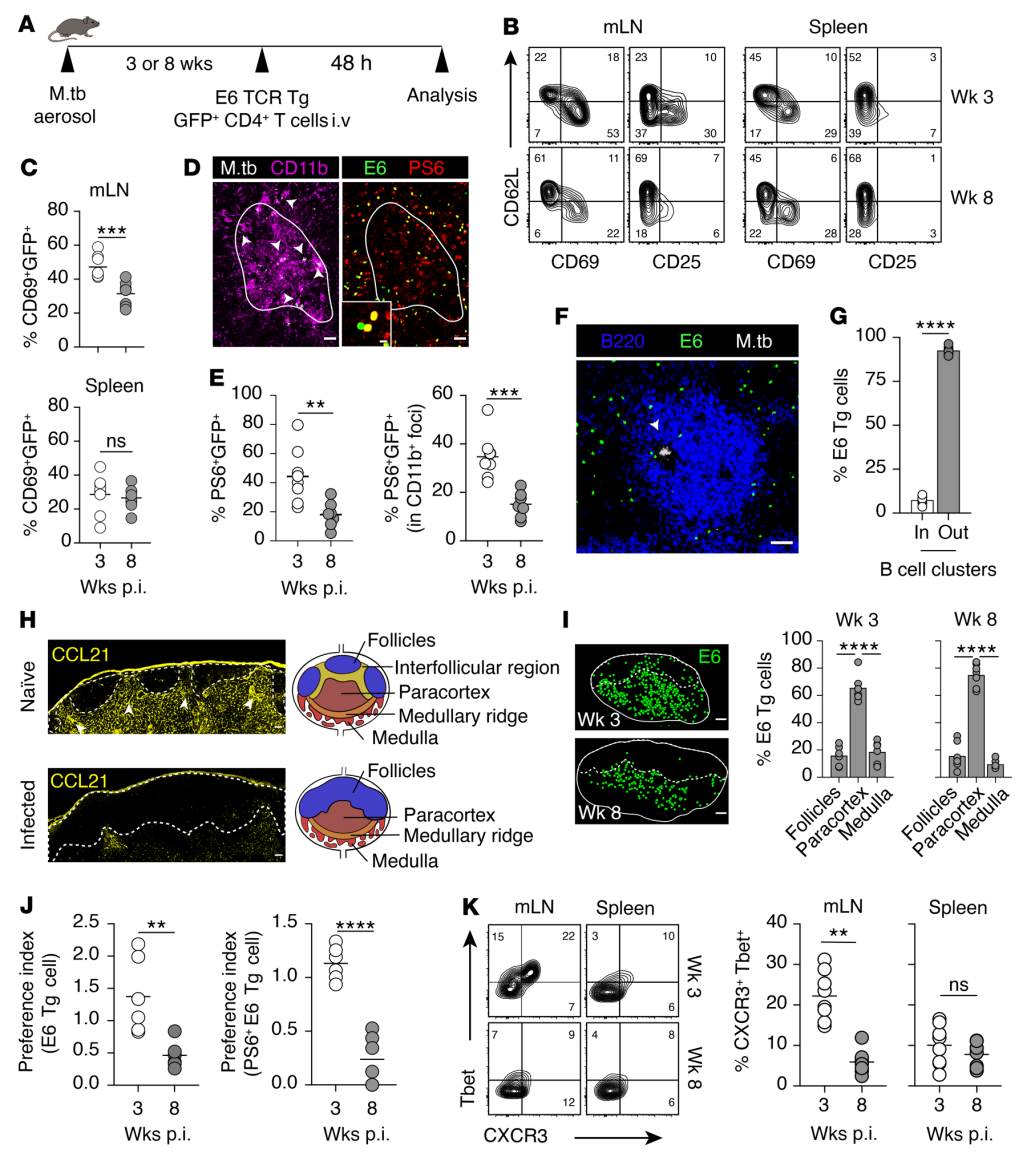
Progressively altered T cell positioning limits CD4^+^ T cell activation. (**A**) Experimental design. (**B**) Expression of activation markers on GFP^+^ transgenic (Tg) cells at weeks 3 and 8 p.i. (**C**) Percentage of CD69^+^ Tg cells in the mLN and spleen. (**D**) Left: Representative image of *M. tuberculosis* bacteria (arrowheads) and CD11b^+^ cells. Right: Images with enlarged view showing p-S6^+^ and p-S6^–^ GFP^+^ cells. Scale bars: 50 μm and 10 μm (inset). White line: foci border. (**E**) Proportion of p-S6^+^ Tg cells in whole mLN section (left) and within *M. tuberculosis*^+^CD11b^+^ foci (right). (**F**) Image of paracortical B cell cluster and position of E6 Tg cells and *M. tuberculosis* foci (arrowhead). Scale bar: 50 μm. (**G**) Percentages of Tg cells localized inside or outside of paracortical B cell clusters. (**H**) IFR in mLNs of naive and 8-week *M. tuberculosis*–infected mice (enlarged regional view of mLNs from Figure 2A). Dashed lines delineate the B cell follicles. Scale bars: 50 μm. Arrowheads indicate the IFR in mLN section of naive mice. Right: Diagrams of LN compartments in naive (top) and *M. tuberculosis*–infected (bottom) mLN. (**I**) Left: Images of mLN showing location of Tg cells (pseudocolored). Scale bars: 200 μm. Right: Quantification of Tg cells in the subcompartments of the mLN as defined in **H**. (**J**) Preference index indicating localization preference of total Tg (left) or p-S6^+^ Tg cells (right) in infected mLN. (**K**) Percentages of T-bet^+^ and CXCR3^+^ in Tg cells. Data in **B**, **C**, **E**, **G**, and **I**–**K** are from 2 independent experiments (6–8 mice per group in total), and images in **D**, **F**, and **H** are representatives from 2 independent experiments with similar results (3–5 mice per group in total). **P < 0.01, ***P < 0.001, ****P < 0.0001 by Student’s t test (**C**, **E**, **G**, **J**, and **K**) or 1-way ANOVA with Tukey’s multiple-comparison test (**I**).

B cell–driven mLN remodeling may impair naive T cell activation during *M. tuberculosis* infection caused by the progressive disruption of the paracortical microenvironment or increased segregation of pathogen-containing APCs by B cells. To examine this hypothesis, we assessed *M. tuberculosis*–specific T cell response in the early and chronic phase of the infection using an adoptive transfer approach. Naive GFP-expressing, ESAT-6_1–20_ epitope–specific T cell receptor–transgenic (TCR-transgenic) CD4^+^ T cells (clone 7) were injected into 3- or 8-week-infected recipient WT mice, and activation of donor GFP^+^ cells, determined by the expression of the early activation markers CD69 and CD25, was analyzed in the mLN and spleen at 48 hours after transfer (Figure 5A). If we had analyzed the cells at a later time point, such as 72 hours, the interpretation of the findings might have been complicated by the dynamic nature of lymphocyte trafficking, as all transferred antigen-specific cells had become activated and divided at least once (Supplemental Figure 5A) and cells in the earlier divisions may have egressed from the LN at this time point. Flow cytometric analysis of mLN cell suspensions showed that the percentages of donor GFP^+^CD4^+^ T cells that upregulated CD69 and CD25 were significantly higher in 3-week-infected recipients than in 8-week-infected animals (Figure 5, B and C). In contrast to the mLN, similar levels of T cell activation were observed in the spleens of week 3 and 8 recipients, suggesting that T cell response to *M. tuberculosis* is differentially regulated in the 2 secondary lymphoid organs. These differences are unlikely due to differential bacterial control in the mLN and the spleens, as the bacterial burdens were comparable at weeks 3 and 8 p.i. in both organs (Supplemental Figure 5B). In addition, it has been demonstrated previously that in contrast to Ag85b, ESAT-6 expression remains stable throughout the course of the infection as seen in the lungs of mice and humans (34, 61, 62). However, we cannot formally exclude the possibility that the difference in T cell activation is due to changes in antigen availability in the mLN of week 3 and week 8 infected mice.

We next visualized the intranodal location of antigen-specific T cells following *M. tuberculosis* infection using the same adoptive transfer model. The activation status of transferred T cells was monitored by the expression of phospho-S6 (p-S6), a marker of TCR engagement (63), which has been successfully used to study T cell activation in imaging studies (38, 64) (Figure 5D). Consistent with our flow cytometry analysis, we found that the percentage of p-S6–expressing antigen-specific T cells quantified across the entire LN section was significantly lower in 8-week-infected than in 3-week-infected recipient mice (Figure 5E). A similar reduction in the activated transferred T cells was also observed at the level of infected myeloid foci (*M. tuberculosis*^+^ and CD11b^+^, as illustrated in Figure 5D) in 8-week-infected recipients. Together, these results suggest that the accumulation of activated *M. tuberculosis*–specific T cells is decreased in the mLN of chronically infected mice.

Considering that the preceding experiments demonstrated disruption of the T cell zone by invading B cell clusters (Figures 1 and 2), we questioned whether antigen-specific T cells would be excluded from these B cell clusters. To assess this hypothesis, we quantified the transferred *M. tuberculosis*–specific T cells in and outside B cell clusters within the paracortex of the infected mLN (Figure 5F and Supplemental Figure 3E) and found that more than 75% of the transferred GFP^+^ T cells were localized outside of B cell clusters (Figure 5, F and G, and Supplemental Figure 3E). This suggests that B cell clusters within the paracortex interfere with the access of antigen-specific T cells to *M. tuberculosis* lesions that could be localized within the clusters.

Previous studies have suggested that, following initial interaction with APCs within the central paracortex, CD4^+^ T cells must migrate to peripheral regions of the LN, IFR, and MR for optimal Th1 differentiation (15, 16, 18). Therefore, we assessed the intranodal location of *M. tuberculosis*–specific T cells in 4 major LN microenvironments: B cell follicles, central paracortex, MR, and the medullary area excluding the IFR. The IFRs are localized between B cell follicles and can be identified by the dense expression of CCL21 (9), which in naive mLN exhibited an organized reticular network that delineated the follicles. However, typical IFRs were lost following *M. tuberculosis* infection, as demonstrated by the expansion of B cell follicles (Figures 1 and 2) and diminished expression of CCL21 (Figure 5H and Supplemental Figure 3F). Consequently, we decided to exclude IFRs from our analysis, as they were no longer discernible following *M. tuberculosis* infection. Importantly, the disruption of IFRs is likely to be a mechanism underlying the impaired T cell activation described above and reported previously (65).

Following adoptive cell transfer, GFP^+^ transgenic T cells were mainly localized in the central paracortex of the recipient mice regardless of the stage of infection (Figure 5I). However, in 3-week-infected recipients, these cells appeared to aggregate in clusters and localize in the outer parts of the paracortex. In contrast, in 8-week-infected recipient mice, the GFP^+^ cells appeared to become dispersed throughout the paracortex. To determine whether peripheralization of transferred CD4^+^ T cells occurs in the infected mLN, we focused on analyzing the cellular movement at the MR, as infected mLN had almost completely lost the IFRs (Figure 5H). We compared the ratio of T cell density in the MR to that in the central paracortex (preference index). A preference index greater than 1 indicates a preferential localization of transferred cells to the MR, whereas less than 1 suggests a preference for the central paracortex (Figure 5J). We observed that TCR-transgenic CD4^+^ T cells transferred into 3-week-infected recipients exhibited a strong preference to migrate to the MR, while the same cells appeared to remain in the central paracortex in 8-week-infected recipient mice. Quantification of activated p-S6^+^GFP^+^ cell localization showed similar results, demonstrating a greater preference to localize to the MR. This analysis suggests that the LN microenvironment impedes the process of CD4^+^ T cell activation in the later stage of the infection (week 8).

Peripheralization of CD4^+^ T cells has been associated with enhanced Th1 differentiation in mice (15). In accordance with this, we found that the percentage of CXCR3^+^T-bet^+^GFP^+^ cells in mLNs of 3-week-infected mice was significantly higher than that in 8-week-infected mice, with no significant differences observed in the spleens (Figure 5K). Taken together, these results suggest that the decrease in T cell peripheralization to the MR at week 8 p.i. is associated with reduced T cell activation and impaired Th1 differentiation.

### Chronic pulmonary M. tuberculosis infection impairs naive CD4^+^ T cell response to an unrelated antigen in mLN.

**Figure 6:**
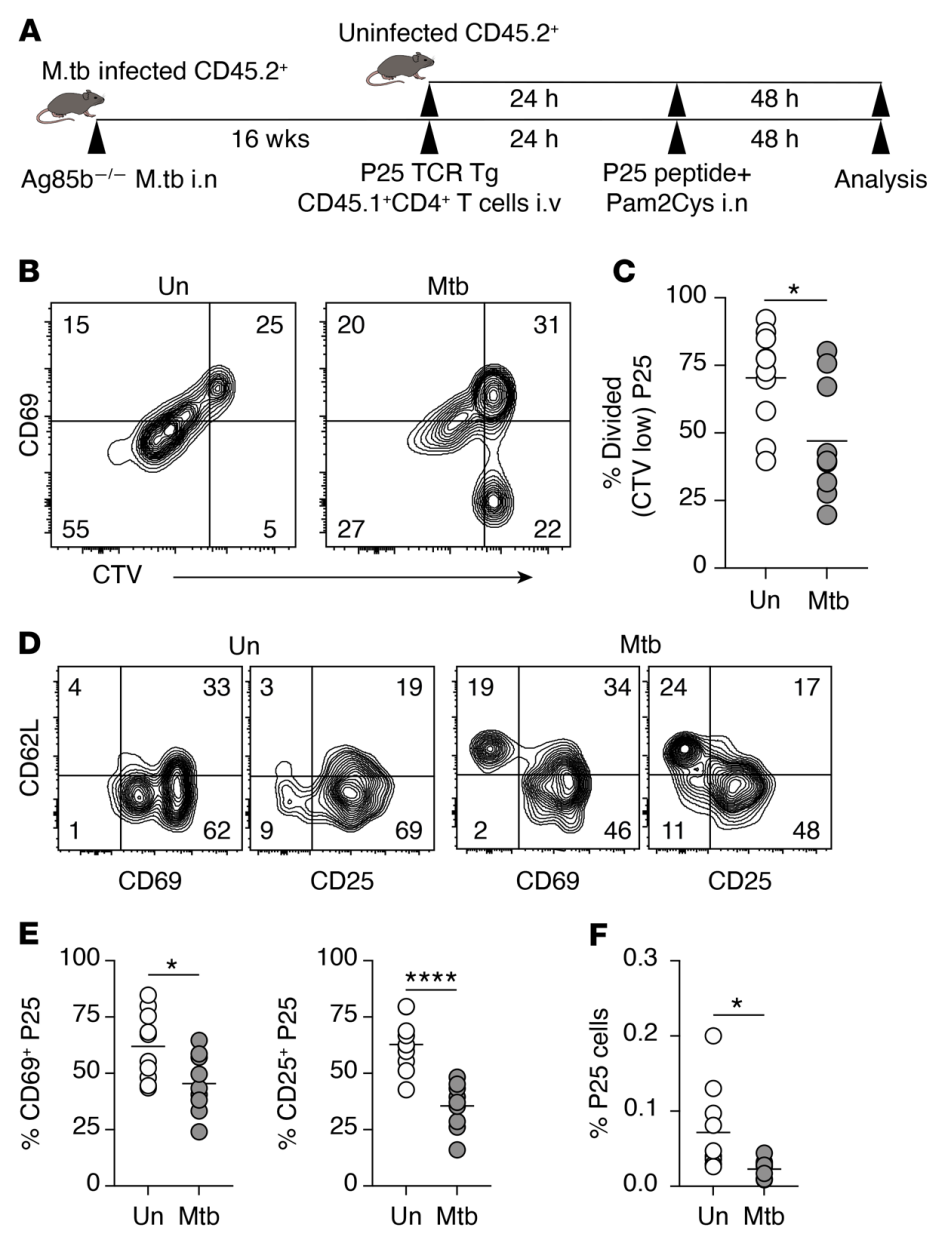
Chronic pulmonary *M. tuberculosis* infection impairs naive CD4+ T cell response to an unrelated antigen in mLN. (**A**) Experimental design. B6 mice were infected with Ag85b*^–/–^* *M. tuberculosis* intranasally (i.n.) or remained uninfected. At 16 weeks p.i., infected and uninfected mice received i.v. CTV-labeled splenocytes from Ag85b-specific P25.CD45.1^+^.*Rag1^–/–^* TCR-Tg mice (~5 × 10^5^ CD4^+^ T cells per recipient). Twenty-four hours later, both groups of mice were inoculated with 10 μg Ag85b_240–254_ peptide together with Pam2Cys adjuvant i.n. The mLNs were collected 48 hours after inoculation. (**B** and **C**) Flow cytometry plots showing the expression of CD69 on CTV-labeled P25 cells (**B**) and summary graph of the proportion of divided P25 cells (CTV low) (**C**) in mLN of uninfected (Un) and infected (Mtb) mice. (**D**) Representative flow cytometry plots showing the expression of CD62L, CD69, and CD25 on P25 cells. (**E**) Summary graphs showing the proportion of CD69^+^ and CD25^+^ P25 cells in mLN of uninfected and *M. tuberculosis*–infected mice. (**F**) The proportion of P25 cells in total CD4^+^ T cells in infected and uninfected mice. Data shown are from 2 independent experiments (*n* = 10 mice per group in total). Statistical differences between groups were determined using Student’s *t* test. *P < 0.05, ****P < 0.0001.

The preceding findings suggested that pulmonary *M. tuberculosis* infection distorts mLN structure and impairs priming of pathogen-specific CD4^+^ T cells. To address whether the LN remodeling process also compromises T cell response to an unrelated antigen, we intranasally infected one group of mice with an *M. tuberculosis* strain that is deficient in a major secreted protein, Ag85b (Ag85b*^–/–^*), while the second group remained uninfected. At 16 weeks p.i., both groups of mice were challenged with Ag85b_240–254_ peptide together with Pam2Cys adjuvant intranasally. Twenty-four hours later, we adoptively transferred CellTrace Violet–labeled (CTV-labeled) Ag85b-specific TCR-transgenic CD45.1^+^CD4^+^ T cells (P25) (i.v.) and assessed the activation and proliferation of the transferred Ag85b-specific T cells 48 hours after transfer (Figure 6A).

By analyzing CTV and CD69 expression, we found that Ag85b-specific CD4^+^ T cells in the *M. tuberculosis*–infected recipients were slower to enter cell division and upregulate CD69 compared with their counterparts in naive recipients (Figure 6, B and C). The donor T cells in *M. tuberculosis*–infected mice also exhibited delayed upregulation of CD25 and downregulation of CD62L (Figure 6, D and E). Consequently, Ag85b-specific CD4^+^ T cells expanded to a significantly lower magnitude in *M. tuberculosis*–infected recipient mice than in naive recipients (Figure 6F), with less than half the percentage of Ag85b-specific cells in infected as compared with uninfected recipients at 48 hours after Ag85b peptide challenge. The percentage of Ag85b-specific cells remained lower in the mLNs of the *M. tuberculosis*–infected mice even when they were challenged with a higher Ag85b_240–254_ peptide dose at 50 μg (data not shown). Together, these findings indicate that modulation of the mLN microenvironment by chronic inflammation leads to suboptimal activation of naive CD4^+^ T cells against unrelated antigens.

## Discussion

LN architecture is essential for the communication between APCs and rare antigen-specific T cells and the subsequent generation of an effective immune response. Previous studies have focused mainly on the characterization of LNs following the single inoculation of model antigen or adjuvant (9, 49) or pathogens that only cause acute infections in mice (19, 20, 22). There is little information regarding the impact of chronic infection on the architectural integrity and, more importantly, the T cell–priming function of the LN. In this study, we show that persistent pulmonary *M. tuberculosis* infection leads to drastic remodeling of the lung-draining mLN. Disproportionate expansion of B cell populations diminishes the IFR and induces the development of ectopic FDCs, which leads to the displacement of CCL21-secreting FRCs and T cells within the paracortex. Functionally, naive CD4^+^ T cell response to both *M. tuberculosis* and unrelated antigens is severely impaired in the mLN of chronically infected mice. These findings reveal new tissue-specific cellular changes in *M. tuberculosis*–infected mice and the impact of chronic inflammation on LN architecture and CD4^+^ T cell function.

Unexpectedly, we found that the development of cell-mediated immune response to *M. tuberculosis* is accompanied by substantial B cell accumulation in the mLN. The function of B cells in *M. tuberculosis* infection is poorly defined. Previous studies focusing primarily on the role of B lymphocytes in bacterial control have generated inconsistent results. B cells have been shown to be inconsequential (66–68) or protective (69–71) in the control of *M. tuberculosis* infection in mice and nonhuman primate models. Our findings show that intranodal B lymphocyte expansion disrupts lymphoid structure and CD4^+^ T cell response, revealing a major regulatory role for B cells in generating T cell responses in the lung-draining mLN. Mechanistically, this regulatory activity of B lymphocytes is dependent on their population size and intranodal localization, and occurs through modulation of the positioning of structural stromal cells.

The expansion of B cell populations and their invasion into the paracortex is associated with the formation of ectopic B cell structures underpinned by CD35^+^CCL21^–^ FDC clusters. Many of these lesions were found to contain *M. tuberculosis* bacteria, particularly during the later phase of the infection. These structures could either contribute to the containment of the infection or aid the survival of the pathogen. However, our B cell depletion experiment results favor the latter possibility. The spatial segregation of *M. tuberculosis*–containing myeloid cell foci from T cells by the ectopic paracortical B cell structures may subvert the development of antimycobacterial Th1 cell responses. Therefore, infection-driven B cell expansion may represent a pathogen evasion strategy facilitating the pathogen’s survival and establishment of persistent infection. Interestingly, B cell–deficient mice were found to have reduced bacterial loads in the spleen but not the lungs in previous studies (72, 73), suggesting that the promycobacterial function of B cells is more profoundly exerted in the secondary lymphoid organs than in nonlymphoid peripheral tissues.

Our data reveal a previously unrecognized mechanism regulating the integrity of the stromal network expressing the T cell–homing chemokine CCL21 during infection. The global paracortical loss of CCL21 expression has been observed previously and attributed to physical destruction of structural cells (46, 74), IFN-γ (19), or Socs3/Smads-dependent (23) suppression induced by *Salmonella* LPS in mice. Although both *Salmonella* and *Mycobacterium* are intracellular bacteria, the latter mechanism is unlikely to contribute in our model, as LPS is not synthesized by *M. tuberculosis* (75). Persistent *M. tuberculosis* infection does not suppress CCL21 expression per se, but rather alters the spatial organization of CCL21-producing cells caused by the development of ectopic CD35^+^FDC^–^ B cell clusters in the paracortex. A previous study with complete Freund’s adjuvant (CFA) immunization has suggested that B cells may have a role in altering the FRC network with disorganization of lymphocytes at the T–B cell border during inflammation (9, 49). Moreover, the CFA-expanded B cell follicles are supported by 2 types of stromal cells: CD21/35^+^ MRCs and CD21/35^–^ VSCs (49, 50). In our study, we demonstrated that paracortical B cell clusters colocalized with CD35^+^ stromal cells that are likely MRCs. MRCs are progenitors of FDCs that have been demonstrated to be the main contributors to FDC expansion during inflammation (50). However, we cannot rule out the potential contribution of VSCs, which have been shown to support B cell expansion by displaying FDC functional properties (49). We propose that the infection-induced expansion of B cell follicles triggers the differentiation of MRCs into FDCs and their invasion of the paracortex along with the expanding B cells.

We demonstrated that modulation of the LN microenvironment by chronic *M. tuberculosis* infection is associated with the impaired generation of CD4^+^ T cell response at this site. The critical importance of the CCL21/CCL19-expressing network in generating a functional T cell response is exemplified in plt/plt mice that lack these T cell–homing chemokines (41). Although adaptive immune responses are generated in plt/plt mice, the altered lymphocyte organization markedly impairs the quality of T cell response (41). Interestingly, a previous study has reported a decline in the activation of *M. tuberculosis*–specific CD4^+^ T cells after week 4 p.i. in mice, and the authors hypothesized that the impairment could be due to disruption in the LN microenvironment (65). Our studies show that the displacement of CCL21-secreting paracortical FRCs leads to the reorganization of the T cell zone and gradual loss of T cell reactivity. We propose that deterrence of T cells from *M. tuberculosis*–infected myeloid foci and displacement of T cells within the paracortex are the key mechanisms regulating the CD4^+^ T cell response to *M. tuberculosis*. Several murine studies have suggested that the initial APC–T cell interaction occurs within the paracortex (15, 76, 77). Following T cell priming, intranodal migration of recently activated cells into specific regions of LN determines T cell fate decision (13, 15, 78). In the case of Th1 cells, trafficking of recently activated CD4^+^ T cells to peripheral regions of LN in a CXCR3-dependent manner is strongly associated with enhanced Th1 differentiation (15). In agreement with these previous studies, we have demonstrated that increased peripheralization of p-S6^+^ T cells was associated with increased *M. tuberculosis*–specific CXCR3^+^ Th1 cells at week 3 compared with week 8 p.i. The impaired CD4^+^ T cell peripheralization in *M. tuberculosis* infection may result from at least 2 B cell–dependent architectural changes: the disappearance of the IFR, and reduced trafficking of activated CD4^+^ T cells to the MR.

The marked organizational remodeling demonstrated in *M. tuberculosis*–infected mLN impairs naive T cell response to not only *M. tuberculosis* antigens but also an unrelated antigen. These findings parallel previous studies in which the loss of lymphoid organization during initial stages of an immune response was shown to render the mice susceptible to secondary infections (19, 22). However, the underlying causes of the observed defects differ significantly, as the host response to the secondary challenge depends on the type of primary infection agent and immune response. Previous studies were performed in models of acute infection, which are starkly different from the *M. tuberculosis* infection model in which the pathogen persists throughout the lifespan of infected mice (30). Furthermore, while immune responses mediated by B cells (22) and CD8^+^ T cells (19) were analyzed in the previous investigations, we examined the antigen-specific CD4^+^ T cell response to an unrelated antigen in chronically infected mice. Given that 10 million people develop active tuberculosis annually (79), it would be interesting to examine whether pulmonary *M. tuberculosis* infection impacts the host response to subsequent exposure to other respiratory pathogens such as influenza, SARS-CoV-2, or airborne allergens.

Our findings demonstrate that B lymphocytes regulate CD4^+^ T cell response by remodeling T cell–homing chemokine–secreting FRC network in the paracortex. We suggest that this B cell–dependent regulatory mechanism plays a dual role in *M. tuberculosis* infection; while allowing *M. tuberculosis* to persist, the impaired Th1 response associated with chronic infection may mitigate the risk of excessive immunopathology in the lungs (80). The cause of increased accumulation of intranodal B cells remains unclear. However, our results suggest that this is most likely independent of Th1 response or enhanced B cell proliferation. One possible explanation could be enhanced B cell recruitment and/or retention, as seen in a subcutaneous murine tumor model (81). Addressing the mechanisms underpinning B cell expansion in the LN in the future may yield new insights relevant to the control of long-term disruption of LN microenvironment by chronic inflammation commonly associated with persistent intracellular pathogen infection and cancer.

## Methods

### Mice.

WT C57BL/6 mice (6–8 weeks of age) were purchased from Australian BioResources. TCR-Tg mice specific for ESAT-6_1–20_:I-A^b^ complexes (clone 7) (26) were bred with GFP-expressing B6 *Rag1^–/–^* mice to generate E6.GFP^+^.*Rag1^–/–^* (82). TCR-Tg mice specific for Ag85b_240–254_:I-A^b^ complexes (P25) (83) were on *Rag1^–/–^* background and expressed CD45.1 (P25.CD45.1^+^.*Rag1^–/–^*). *Ifnar1^–/–^*, *Ifngr1^–/–^*, and *Tnfr1^–/–^* mice as well as TCR-Tg mouse lines were bred and maintained at the Centenary Institute Animal Facility.

### Generation of chimeric mice.

WT C57BL/6 and *Ifngr1^–/–^* mice were irradiated with 10 Gy. Irradiated mice were reconstituted with a total of 2 × 10^6^ bone marrow cells from WT mice. Mice received antibiotic-supplemented (trimethoprim/sulfamethoxazole) drinking water for 8 weeks after irradiation. Mice were used 12 weeks after bone marrow reconstitution.

### M. tuberculosis culture, infection, and peptide challenge.

WT *M. tuberculosis* (strain H37Rv) and Ag85b*^–/–^* *M. tuberculosis* were grown to log phase at 37°C in Middlebrook 7H9 broth (BD Biosciences) supplemented with 0.5% (vol/vol) glycerol, 0.05% (vol/vol) Tween-80 (Sigma-Aldrich), and 10% albumin-dextrose-catalase. *M. tuberculosis* (clinical isolate MT103) deficient in Ag85b was constructed using the pYUB854-85KO and pJV53 plasmids (84), as described previously for BCG (85). Mice were infected with H37Rv via aerosol route using a Middlebrook airborne infection apparatus (Glas-Col) with an infective dose of approximately 100 viable bacilli per lung. For infection with Ag85b*^–/–^* *M. tuberculosis* strain, mice were infected via the intranasal route (i.n.) (institutional biosafety regulations prohibit the delivery of genetically modified bacteria with the airborne infection apparatus), with an infective dose of approximately 100 viable bacilli per lung. Colony-forming units in tissue homogenates were quantified using Middlebrook 7H11 Agar (BD Biosciences) supplemented with oleic acid–albumin–dextrose–catalase and 0.5% glycerol. Where indicated, mice were challenged i.n. with 10 μg or 50 μg of peptide P25 (Ag85b_240–254_) with 8 μg of Pam2Cys-SK4-PEG(OH) (provided by R. Payne) (86) in 20 μL PBS.

### Depletion of lymphocytes.

To deplete CD4^+^ T cells, mice received intraperitoneal injections of 500 μg of anti-CD4 mAb GK1.5 or InVivoPlus Mouse IgG2b Isotype control (Bio X Cell) twice a week for 4 weeks, starting from 4 weeks p.i. For the depletion of B cells, mice received intraperitoneal injections of 250 μg rituximab (anti-CD20 mAb) (a gift from Genentech) or InVivoPlus Mouse IgG2a Isotype control (Bio X Cell) twice a week. Anti-CD20 mAb injections were administered either on day 1 p.i. or starting from week 12 p.i. to analyze mLNs at weeks 3 and 16 p.i., respectively.

### Preparation of single suspension from tissues.

mLNs, spleens, and lungs were collected in RPMI supplemented with 2% FCS for flow cytometric analysis. The caudal mLN in mice is a single LN localized in the caudal mediastinum ventral to the esophagus (87). LN and spleens were mechanically dissociated through 70 μm strainers. Lungs were perfused with PBS by cardiac cannulation and collected in 2 mL RPMI medium supplemented with 0.1 mg/mL DNase I (Sigma-Aldrich) and 10 U/mL collagenase type I (Sigma-Aldrich). Lungs were dissociated using GentleMacs Dissociator (Miltenyi Biotec) and incubated for 30 minutes at 37°C. Lungs were then further dissociated with GentleMacs Dissociator and filtered through a 40 μm strainer. Single-cell suspensions were treated with ACK lysis buffer to remove erythrocytes. Cells were washed with RPMI medium supplemented with 2% FCS before viable cells were counted using trypan blue exclusion on a hemocytometer.

### Isolation, CTV labeling, and adoptive transfer of TCR-Tg CD4^+^ T cells.

Splenocytes from E6 or P25 mice were collected and processed to single-cell suspensions. P25 cells were labeled with 5 μM CellTrace Violet (CTV; Thermo Fisher Scientific) in PBS supplemented with 0.1% FCS for 20 minutes in the dark at 37°C. CTV labeling was terminated by addition of cold RPMI medium supplemented with 10% FCS. All cell suspensions were washed and resuspended in PBS before cell transfer. Where indicated, approximately 2 × 10^6^ E6 cells (containing ~2 × 10^5^ CD4^+^ T cells) or approximately 9 × 10^6^ P25 cells (containing ~5 × 10^5^ CD4^+^ T cells) were injected i.v. into WT mice.

### Flow cytometry.

Spleens, LNs, and lungs were stained in FACS wash (2% FCS/PBS/2 mM EDTA) containing surface receptor antibody mixture, Fc block (2.4G2, BD Biosciences), and Live/Dead fixable blue dead cell stain (Thermo Fisher Scientific) for 30 minutes at 4°C in the dark. The following antibodies and clones were used for the detection of surface markers: CD4 (RM4-5), B220 (RA3-6B2), CD62L (MEL-14), CD44 (IM7), CD25 (PC61), and CD69 (H1.2F3) (all from BD Biosciences) and CXCR3 (CXCR3-173, BioLegend). For detection of the intracellular transcription factor T-bet, surface-stained cells were fixed with 100 μL Cytofix fixation buffer (BD Biosciences) for 15 minutes at 4°C. Cells were washed in 1× permeabilization buffer (BD Biosciences). Cells were incubated for 1 hour at 4°C in 1× permeabilization buffer containing T-bet (4B10, Thermo Fisher Scientific). For detection of antigen-specific CD4^+^ T cells, single-cell suspensions were incubated in FACS wash (2% FCS/PBS/2 mM EDTA) containing ESAT-6_4–17_:I-A^b^ (NIH Tetramer Core Facility, Atlanta, Georgia, USA) and Fc block (2.4G2, BD Biosciences) for 1 hour at 37°C prior to antibody staining of surface markers. Cells were washed in 1× permeabilization buffer and resuspended in FACS buffer before acquisition. All flow cytometry data acquisition was performed on an LSR II using FACSDiva software (BD Biosciences), and analysis was performed using FlowJo version 10.8.0 (BD Biosciences).

### LN collection and processing for imaging.

Single mLNs from individual mice were fixed in 1% paraformaldehyde (Alfa Aesar) at 4°C for 24 hours. Fixed tissues were then placed in 30% (vol/vol) sucrose (Sigma-Aldrich) in PBS. Twenty-four hours later, tissues were frozen in optimal cutting temperature compound (VWR Chemicals). Tissues were sectioned at 10–14 μm on a Shandon Cryotome E (Thermo Fisher Scientific). Sections were blocked and permeabilized with 3% normal goat serum (NGS) and 0.1% Triton X-100 diluted in PBS (Cell Signaling Technology) for 30 minutes at room temperature. Sections were then stained with the appropriate primary antibodies diluted in 3% NGS in PBS for 2 hours at room temperature or overnight at 4°C. Sections were washed in 1× Tris-buffered saline with 0.5% Tween-20 (TBST) before staining with secondary antibodies diluted in 3% NGS in PBS for 1 hour at room temperature. The sections were washed 3 times in 1× TBST and subsequently mounted with ProLong Gold Antifade (Thermo Fisher Scientific). The following antibodies (clone number, source) were used in this study: CD3 (17A2, BioLegend), B220 (RA3-6B2, BD Biosciences), anti–purified protein derivative (polyclonal ab905, Abcam), CD11b (M1/70, BioLegend), CD169 (3D6.112, eBioscience), Lyve-1 (polyclonal ab33682, Abcam), CCL21/6Ckine (polyclonal, R&D Systems), anti–reticular fibroblasts (ER-TR7, Abcam), podoplanin (8.1.1, BioLegend), p-S6 (2F9, Cell Signaling Technology), biotin–anti-CD35 (8C12, BD Biosciences), goat anti-rabbit Alexa Fluor 647 (polyclonal, Thermo Fisher Scientific) or Alexa Fluor 555 (polyclonal, Thermo Fisher Scientific), donkey anti-rat Alexa Fluor 488 (polyclonal, Thermo Fisher Scientific), and streptavidin–Alexa Fluor 555 (BD Biosciences).

Sections were imaged on a DeltaVision Personal Imaging System (GE Healthcare). Image analysis was performed using Fiji software version 2.0.0-rc-69/1.53g (NIH Research Services).

### Image analysis.

For each mLN, 4 stitched images of non-consecutive sections (>30 μm apart, to obtain distinct tissue planes) were examined per mouse, and 1 representative image from each animal was selected for downstream analysis. Regions of interest (ROIs), including the paracortex, B cell follicles, and the medulla, were identified using immunostaining of B cells (B220^+^), T cells (CD3^+^), and lymphatic vessels (Lyve-1^+^), respectively, on the same or consecutive LN sections. The B cell follicles were defined as the region in the cortex of the LN. The medulla was defined as the region in the basal area of the LN based on Lyve-1 staining and histology of medullary cords. The paracortex was defined based on CD3 staining and as the area between the cortical follicles and the medulla. The MR was identified as the area 80 μm away from the medulla. The intranodal localization of infected foci (CD11b^+^*M. tuberculosis*^+^) and transferred E6 cells (GFP^+^ or p-S6^+^GFP^+^) was counted on stitched entire LN images. The number of infected foci inside B cell clusters (surrounded by ≥10 μm B220^+^ cells) was counted within the paracortex of whole LN sections.

Transferred TCR-Tg cells found within or outside of B cell clusters were counted in B220^+^ cell aggregates within the paracortex of whole LN sections. All cell counting was performed using the cell counter plug-in in Fiji software. To determine the fluorescent intensity profile, paracortical areas containing B cell clusters were selected, and fluorescence intensity was measured from the cortex (outer portion of LN) to the medulla, using the plot profile plug-in in Fiji software. To calculate preference index, the area of ROIs — paracortex and MR — was measured in square micrometers on Fiji software. Transferred E6 cells (GFP^+^ or p-S6^+^GFP^+^) were counted within each ROI, and the density per area was calculated using the following formula: [cell number in MR/MR area size (μm^2^)]/[cell number in paracortex/paracortex area size (μm^2^)].

### mRNA preparation and quantitative reverse transcriptase PCR.

LNs were collected and submerged in RNAlater (Ambion) for 24 hours (4°C) prior to long-term storage at –80°C. RNA was purified from LNs using TRIsure (Bioline) per the manufacturer’s instructions. RNA (2 μg) was reverse-transcribed using the Tetro cDNA synthesis kit with random primers according to the manufacturer’s instructions (Bioline).

Quantitative reverse transcriptase PCR (qRT-PCR) was performed using SensiFast SYBR Green (Bioline) on a Roche LightCycler 480. Data are expressed as fold change over uninfected control LNs. Data were calculated by ΔΔCt method using 18S as the reference gene. The following forward (Fw) and reverse (Rv) qRT-PCR primers were used in this study: *18S* Fw GTAACCCGTTGAACCCCATT, Rv CCATCCAATCGGTAGTAGCG; IFN-γ Fw ACAATGAACGCTACACACTGCAT, Rv TGGCAGTAACAGCCAGAAACA; CCL21 Fw CTGCAAGAGAACTGAACAGAC, Rv CTTCTGACTCTCTAGGTCTAC.

### Antibody ELISA.

Serum samples were collected before and at weeks 3, 8, and 10 after *M. tuberculosis* infection. Microtiter 96-well plates (Costar) were incubated overnight with 5 μg/mL *M. tuberculosis* culture filtrate (CFP; BEI Resources) or 5 μg/mL whole-cell lysate (WCL; BEI Resources) in carbonate buffer at room temperature. Plates were then blocked for 2 hours with 3% BSA in PBS, and serially diluted serum samples were incubated for 45 minutes at 37°C. Plates were washed, and biotinylated polyclonal goat anti-mouse IgG2c (1:10,000; Abcam, catalog ab97253), goat polyclonal anti-mouse IgG1 (1:50,000; Abcam, catalog ab97238), or goat polyclonal anti-mouse IgG (1:100,000; Abcam, catalog ab6788) was added for 1 hour at room temperature. After incubation with streptavidin-HRP (1:30,000; Abcam, catalog ab7403) for 30 minutes at room temperature, binding was visualized by the addition of tetramethyl benzene in phosphate citric buffer (Sigma-Aldrich). The reaction was stopped with the addition of H_2_SO_4_, and absorbances were measured at 450 nm by a Tecan plate reader. Endpoint titers were estimated by the 5-polynomial sigmoidal curve of each sample interpolated with the threshold of the average of the negative controls ± 3 standard deviations.

### Statistics.

Statistical analysis was performed using GraphPad Prism 9 software (GraphPad Software). Differences between 2 groups were analyzed using unpaired 2-tailed Student’s *t* test, and differences between multiple groups were analyzed using 1-way ANOVA with Tukey’s multiple-comparison or Kruskal-Wallis test, as indicated in the figure legends. Results with a *P* value less than 0.05 were deemed statistically significant.

### Study approval.

All murine experiments were conducted with the approval of the Sydney Local Health District Animal Welfare Committee (protocol 2015-037 and 2020-007).

## Author contributions

LD and CGF conceptualized the study. LD, NDB, and CC performed experiments. LD and CGF wrote the original draft of the manuscript. All authors contributed to the review and editing of the manuscript. CGF, XC, and WJB acquired funding. YC, XC, WJB, and JAT provided resources. CGF supervised the study.

## Supplementary Material

Supplemental data

## Version 1 11/01/2022

Electronic publication
