# Supplementary material for: Stromal structure remodeling by B lymphocytes limits T cell activation in lymph nodes of Mycobacterium tuberculosis–infected mice
Source: J Clin Invest. 2022 Nov 1;132(21):e157873. doi: 10.1172/JCI157873 (PMC9621141; doi:10.1172/JCI157873)
Supplement: Supplemental data [file jci-132-157873-s042.pdf]

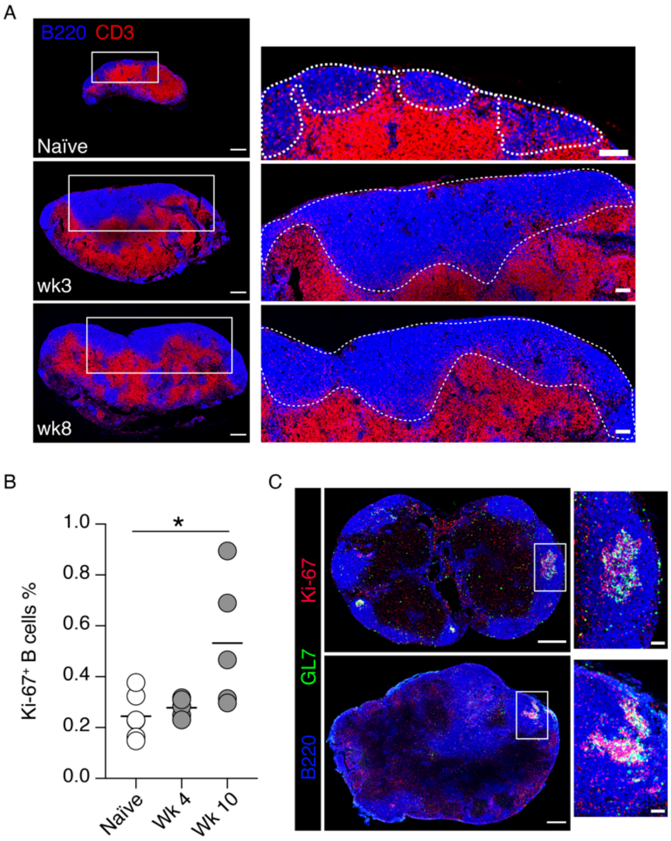

Supplementary Figure 1. B cell expansion, proliferation, and germinal center formation in the lung-draining mLN of M.tb-infected mice.

(**A**) Enlarged view of cortical follicles in mLN from naïve, wk 3- and wk 8-infected WT mice from Fig.1C. The tissues were stained for B220 to detect B cells and CD3 to detect T cells. The dashed lines delineate the cortical follicles. Enlarged images (right panel) of the boxed areas in the left panel show individual cortical follicles in naïve mLN, and partially or fully merged B cell follicles in M.tb infected mLNs. Scale bar, 300 μm (left) and 100 μm (right, enlarged images). (**B**) Ki-67 expression on B220^{+} cells analysed by flow cytometry in LNs of naïve and M.tb infected mice at the indicated time points. (**C**) Stitched images of LN sections from naïve and wk8 M.tb-infected mice stained for Ki-67, GL7, and B220. Scale bar, 300 μm. Enlarged view of the boxed area in the left panels are shown in the right panels to illustrate germinal centre development in B cell follicles. Scale bar, 60 μm. Data in (**A and C**) are representative of more than 5 independent experiments with similar results (n= 5 mice/group). Data in (**B**) are representative of three independent experiments (n= 5 mice/group). Statistical analysis in (**B**) was determined using Student's *t* test. *$p < 0.05$.

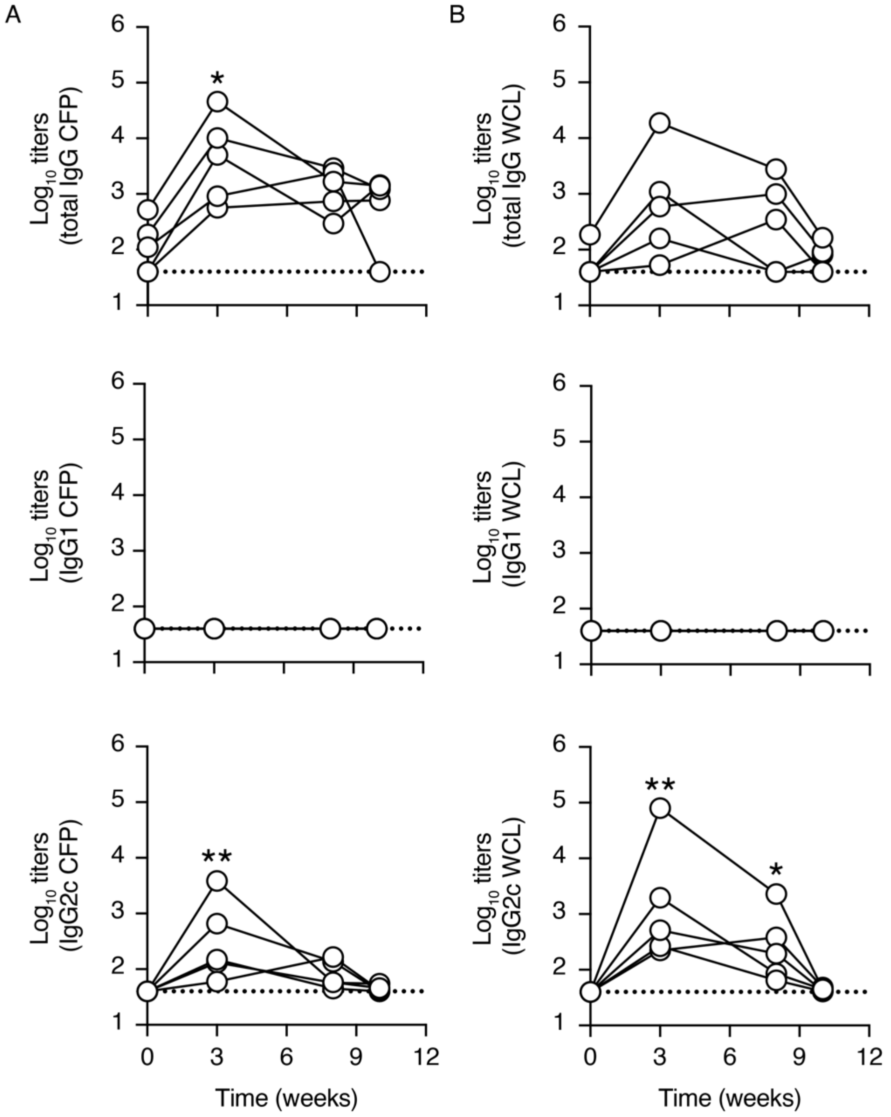

Supplemental Figure 2. Kinetics of pathogen-specific total IgG, IgG1, and IgG2c antibody response in M.tb-infected mice.

(**A** and **B)** Serum was collected from WT B6 mice before and following M.tb infection at the indicated time points. CFP (**A**) and WCL (**B**) specific total IgG, IgG2c and IgG1 were determined by ELISA. The data are presented as titres estimated by the sigmoidal curve of each sample interpolated with the threshold of the negative control samples ±3 standard deviations. Dashed line represents the limit of detection. Each line represents an individual mouse (n=5 mice). Statistical differences between two groups were determined using one-way ANOVA with Kruskal-Wallis test multiple comparisons test. *$p < 0.05$, **$p<0.01$.

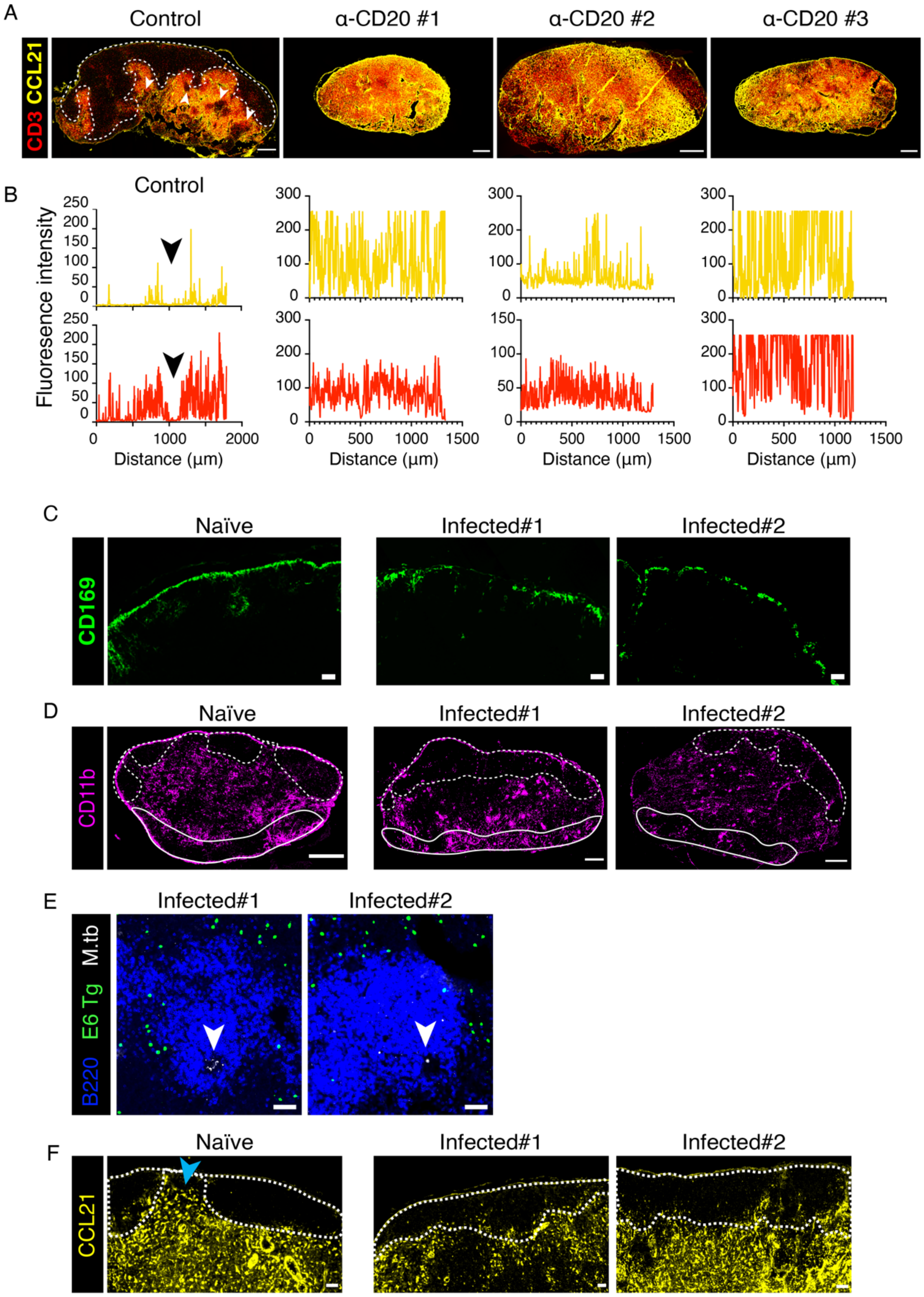
A
Control
α-CD20 #1
α-CD20 #2
α-CD20 #3
CD3 CCL21
B
Control
Fluoresence intensity
Distance (µm)
C
Naïve
Infected#1
Infected#2
CD169
D
Naïve
Infected#1
Infected#2
CD11b
E
Infected#1
Infected#2
B220 E6 Tg M.tb
F
Naïve
Infected#1
Infected#2
CCL21

Supplemental Figure 3. Images of experimental replicates.

(**A**) Representative images of CCL21 and CD3 in mLN of isotype control and anti-CD20 mAb treated M.tb-infected mice. Arrowheads in the control mouse image point to paracortical region occupied by B cell clusters (not shown). Scale bar, 300 μm. (**B**) Histograms representing the average fluorescence intensity of CCL21 and CD3 along the cortex-medullary axis of isotype control and anti-CD20-treated mice from (**A**). Immunofluorescence images of mLN from naïve and wk8 M.tb-infected mice stained for (**C**) CD169 (scale bar, 50 μm) and (**D**) CD11b. Scale bar for naïve and infected mLN is 300 μm. Dashed white line delineates B cell follicles, and solid white line defines the medulla. (**E**) Images of paracortical B cell cluster, E6 Tg cells and M.tb foci (indicated with arrowhead) from the mLN of two individual M.tb-infected mice. Scale bar, 50 μm. (**F**) Inter-folicular regions (IFR) in mLN sections from naïve and 8 wk M.tb-infected mice defined by CCL21 staining. Dashed lines delineate the B cell follicles. Scale bar, 50 μm. Arrowhead indicate the IFR in mLN section of naïve mice. Data shown are images from individual mice and representative of at least two independent experiments (n=3-9 mice/group).

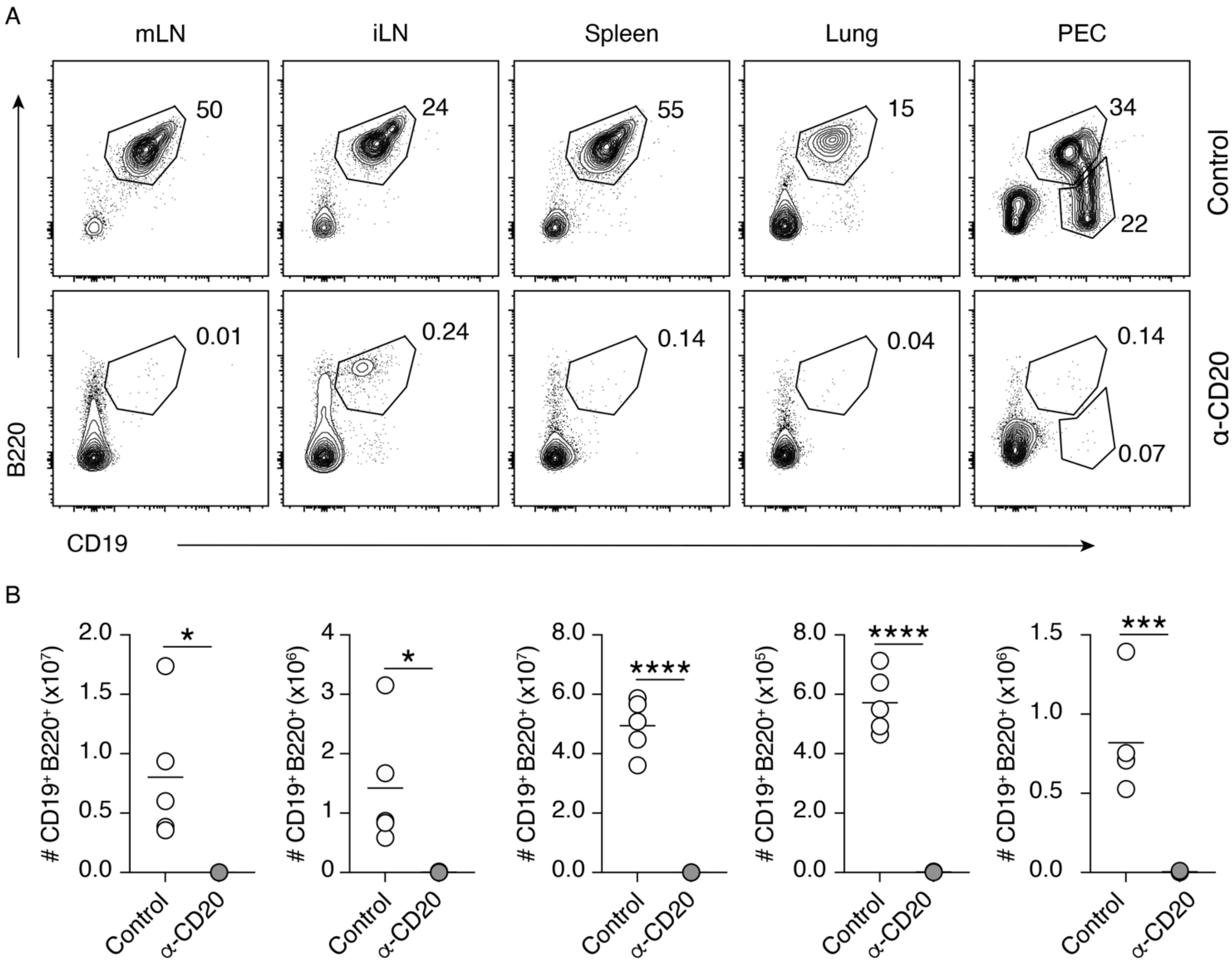

Supplemental Figure 4. Verification of B cell depletion following anti-CD20 treatment in different organs. M.tb-infected mice were injected i.p. with either 250 µg mouse IgG2a isotype control or anti-CD20 antibody twice weekly for 3 weeks and percentage of B cells was analyzed using flow cytometry. (**A**) Representative FACS plots showing the percentage of $B220^{+}CD19^{+}$ B cells in all sites and $B220^{low}CD19^{+}$ B1 cells in peritoneal cavity in treated and non-treated groups. (**B**) Summary graphs of the number of $B220^{+}CD19^{+}$ B cells in control and anti-CD20 treated mice. Data shown are representative of at least two independent experiments with similar trends (n= 5 mice/group). Statistical analysis was determined using Student's *t* test. *$p < 0.05$, ***$p < 0.001$, **** $p < 0.0001$

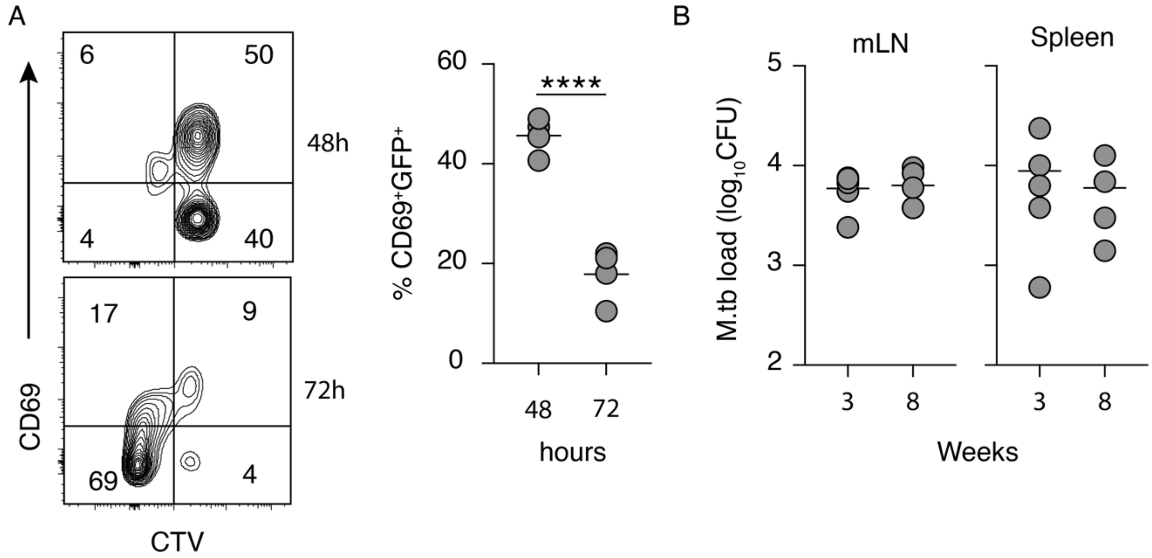

Supplemental Figure 5. Kinetics of CD69 and CTV expression on transferred M.tb-specific CD4$^+$ T cells and bacterial burdens in secondary lymphoid organs.

(A) FACS plots showing the expression of CD69 on CTV-labelled GFP$^+$ ESAT6-specific Tg cells at 48 and 72 h post-transfer in the mLN of wk3 M.tb-infected mice (left panel). Summary graph (Right panel) of the proportion of CD69$^+$ Tg cells in the mLN. (**B**) Bacterial loads in the mLN and spleens of M.tb-infected mice at week 3 and 8 p.i. measured as CFU. Data shown are representative of two independent experiments with similar trends (n=4-5 mice/group). Statistical analysis was determined using Student's *t* test. **** $p < 0.0001$.
